# Exploring patterns of response across the lifespan: the Cambridge Centre for Ageing and Neuroscience (Cam-CAN) study

**DOI:** 10.1186/s12889-018-5663-7

**Published:** 2018-06-19

**Authors:** Emma Green, Holly Bennett, Carol Brayne, Cam-CAN, Lorraine K. Tyler, Carol Brayne, Edward T. Bullmore, Andrew C. Calder, Rhodri Cusack, Tim Dalgleish, John Duncan, Richard N. Henson, Fiona E. Matthews, William D. Marslen-Wilson, James B. Rowe, Meredith A. Shafto, Karen Campbell, Teresa Cheung, Simon Davis, Linda Geerligs, Rogier Kievit, Anna McCarrey, Abdur Mustafa, Darren Price, David Samu, Jason R. Taylor, Matthias Treder, Kamen Tsvetanov, Janna van Belle, Nitin Williams, Lauren Bates, Tina Emery, Sharon Erzinçlioglu, Andrew Gadie, Sofia Gerbase, Stanimira Georgieva, Claire Hanley, Beth Parkin, David Troy, Tibor Auer, Marta Correia, Lu Gao, Emma Green, Rafael Henriques, Jodie Allen, Gillian Amery, Liana Amunts, Anne Barcroft, Amanda Castle, Cheryl Dias, Jonathan Dowrick, Melissa Fair, Hayley Fisher, Anna Goulding, Adarsh Grewal, Geoff Hale, Andrew Hilton, Frances Johnson, Patricia Johnston, Thea Kavanagh-Williamson, Magdalena Kwasniewska, Alison McMinn, Kim Norman, Jessica Penrose, Fiona Roby, Diane Rowland, John Sargeant, Maggie Squire, Beth Stevens, Aldabra Stoddart, Cheryl Stone, Tracy Thompson, Ozlem Yazlik, Dan Barnes, Marie Dixon, Jaya Hillman, Joanne Mitchell, Laura Villis, Fiona E. Matthews

**Affiliations:** 1 Department of Public Health and Primary Care, Cambridge Institute of Public Health, Cambridge, UK; 2 Cambridge Centre for Ageing and Neuroscience (Cam-CAN), University of Cambridge and MRC Cognition and Brain Sciences Unit, Cambridge, UK; 3 MRC Biostatistics Unit, Cambridge Institute of Public Health, Cambridge, UK; 4 Institute of Health and Society, Faculty of Medicine, Newcastle University, Newcastle, UK; 5 Institute of Public Health, Forvie Site, University of Cambridge School of Clinical Medicine, Box 113 Cambridge Biomedical Campus, Cambridge, CB2 0SR UK

**Keywords:** Non-participation, Epidemiological study, Lifespan, Ageing, Neuroscience, Cognition

## Abstract

**Background:**

With declining rates of participation in epidemiological studies there is an important need to attempt to understand what factors might affect response. This study examines the pattern of response at different adult ages within a contemporary cross-sectional population-based cohort, the Cambridge Centre for Ageing and Neuroscience (Cam-CAN).

**Methods:**

Using logistic regression, we investigated associations between age, gender and Townsend deprivation level for both participants and non-participants. Weighted estimates of the odds ratios with confidence intervals for each demographic characteristic were calculated. Reasons given for refusal were grouped into three broad categories: ‘active’, ‘passive’ and illness preventing interview.

**Results:**

An association of age and participation was found, with individuals in middle age groups more likely to participate (age group 48–57 OR: 1.8, 95% CI: 1.5–2.2 and age group 58–67 OR: 2.1, 95% CI: 1.7–2.4). Overall, there was no difference in participation between men and women. An association with deprivation was found, with those living in the most deprived areas being the least willing to participate (fifth quintile OR: 0.6, 95% CI: 0.5–0.7). An interaction between age and gender was found whereby younger women and older men were more likely to agree to participate (*p* = 0.01).

**Conclusion:**

Our findings highlight some of the factors affecting recruitment into epidemiological studies in the UK and suggest that targeted age-specific recruitment strategies might be needed to increase participation rates in future cohort investigations.

**Electronic supplementary material:**

The online version of this article (10.1186/s12889-018-5663-7) contains supplementary material, which is available to authorized users.

## Background

A principal goal of epidemiological studies is to recruit representative samples so research outcomes can be generalised back to a known population. In attempting to achieve such representation and generalisability, it is crucial that researchers encourage a high response rate from potential participants to minimise the risk of non-response bias. Non-response bias occurs when differences exist between individuals choosing to participate in research compared to individuals declining participation. Such differences could lead to inaccurate research conclusions of true patterns of association.

Evidence shows participation in epidemiological studies has been decreasing over time [1–3]. Even studies appearing to report relatively stable response rates over time have observed increases in refusals to participation in more recent years [4], having implications on how many individuals are needed to achieve the target study sample size along with generalisability of findings. Even though response rates vary extensively across studies [5] and this is known to have an impact on interpretation, a review of publications in high impact journals found that a considerable proportion of epidemiology studies fail to report any information regarding response rates [6].

When response information is reported, findings generally seem to suggest women are more likely to participate than men [5, 7, 8]. However, reports vary when examining response across age groups. Some studies find participation rates rise with increasing age [7, 8], whilst others observe younger adults are more likely to consent to taking part [9]. An examination of patterns of consent of over 25,000 individuals across seven epidemiological studies reported associations with both age and gender. A similar pattern emerged across all studies with lower response rates in younger people and in men [10]. Drawing comparisons across studies to gain an accurate reflection of response rates proves difficult due to variations in study design and methods of contacting potential participants.

The cross-sectional Cambridge Centre for Ageing and Neuroscience (Cam-CAN) study was developed to examine associations between epidemiological and cognitive neuroscience data across the adult lifespan to identify factors linked with successful ageing (including neuroimaging). The study design of Cam-CAN provides an interesting opportunity to explore a wealth of behavioural and neuroimaging data across the lifespan from age 18 upwards. Furthermore, as the design employed a sample of individuals drawn from the general population this allowed for a valuable and rare source of neuroimaging data to be collected from a non-volunteer cohort. The design involved a home interview which implemented an inclusion and exclusion criteria to select eligible individuals to proceed into the detailed testing and neuroimaging phases of the project.

Here, we investigate the pattern of response from individuals invited to participate in the Cam-CAN study and examine the reasons given for refusal. We will examine how these factors differ by gender, age and postcode deprivation level (the postal code adopted in the UK which designates a geographical area where a number of addresses can be found).

## Methods

### Identification of study sample

The study population consisted of individuals living within Cambridge City in the United Kingdom. The Primary Care Trust (PCT) was the UK’s primary care system in which almost all UK residents are registered on a largely geographical basis. PCT registers formed the sampling frame for the Cam-CAN study in a two-stage identification process. Initially, anonymised PCT records were requested on individuals aged 18 years and over who were registered across the 14 recruited general practitioner (GP) surgeries. At this stage, records received were restricted to gender, month and year of birth, GP practice and first five digits of postcode. A random sample stratified by age was then drawn and identifying details (registered GP, full name and address) of the selected patients were provided to the study team via the PCT.

### Recruitment of study sample

Lists containing contact details held for the selected individuals were sent periodically to the general practices involved who were asked to confirm individuals were still registered and that the personal details were in agreement with their records. General practices were asked to exclude term-time only students and anyone who they perceived could not speak English to a sufficient level to undertake the interview. General practices were asked to identify any potential participants they believed would be inappropriate to approach, such as those terminally ill or those indicating they did not wish to be approached for involvement in research.

Non-excluded individuals received an invitation letter signed by their GP and an information sheet describing the aims and nature of the Cam-CAN project. Potential participants received details of how they could contact the study team to either provide further contact information to aid the research interviewer when visiting or to decline participation. It was foreseen that letters would unavoidably be sent to a small number of individuals who would lack capacity to consent to participating in the study. In such cases, information was provided for relatives and carers in the information sheet regarding contact details of the study team.

Individuals who did not provide further contact information (such as email address or phone number) and those who had not actively refused were given a minimum time period of 3 weeks before a research interviewer visited, with up to three attempts to find the individual at home. At this visit interviewers discussed details of the study and answered questions regarding involvement. With the agreement of the participant, a convenient place and time was arranged to conduct the interview. For individuals declining to be involved, a reason was sought and later coded into refusal categories.

Research interviewers were trained in the Mental Capacity Act (2005) and assessed at approach if an individual could sufficiently understand what their involvement in the project would mean. If assumed the individual lacked capacity to consent - either by the interviewer on approach or via contact from a relative or carer expressing this opinion, a request was made to undertake an informant interview with someone who could provide information about the selected individual on their behalf. Due to logistical difficulties only a few of these interviews were undertaken and are not discussed further in this paper.

### Research interview

Full written informed consent was obtained prior to conducting the interview. Research interviewers aimed to complete the computerised interview in under 2 h. The interview covered socio-demographics, lifestyle and health, along with cognitive assessment questions. The computerised program gave an immediate indication of eligibility for the more detailed phases of the project (including imaging sessions) based on the responses given. Please refer to the Cam-CAN protocol paper for full details of the question topics, tasks asked and eligibility exclusions [11].

### Quality control of the study

Research interviewers underwent an extensive training course covering all aspects of the interview and safeguarding matters such as lone working within the community. Checks were performed at regular intervals whilst fieldwork was ongoing by assessing tape-recorded interviews and offering re-training to interviewers wherever necessary to ensure standardised interviewing.

### Data management and statistical analysis

Data checking and cleaning was conducted on an on-going basis with the dataset prepared in STATA 12.1 format. The data are released in versions, with version 1 used in this analysis. Logistic regression was chosen to investigate associations between age, gender and Townsend deprivation level [12], with the binary outcome participation versus non-participation. An interaction between age and sex was also tested. These variables were selected as they were available for both participants and non-participants. Estimates of odds ratios with confidence intervals for each demographic characteristic were calculated. Age was split into eight decile age groups (18–27, 28–37, 38–47, 48–57, 58–67, 68–77, 78–87 and 88+) and used as a categorical variable in this analysis. The Townsend deprivation index was split into quintiles (based on the complete eligible population sample). Refusal reasons recorded by the study team were mapped into three broad categories: i) ‘active’ refusals whereby participants expressed the refusal themselves; ii) ‘passive’ refusals indicated by a proxy and iii) illness preventing interview including GP declining permission to contact participants or self/proxy reported illness.

### Ethical approval

Ethical approval for the study was obtained from the Cambridgeshire 2 (now East of England – Cambridge Central) Research Ethics Committee.

## Results

**Fig. 1:**
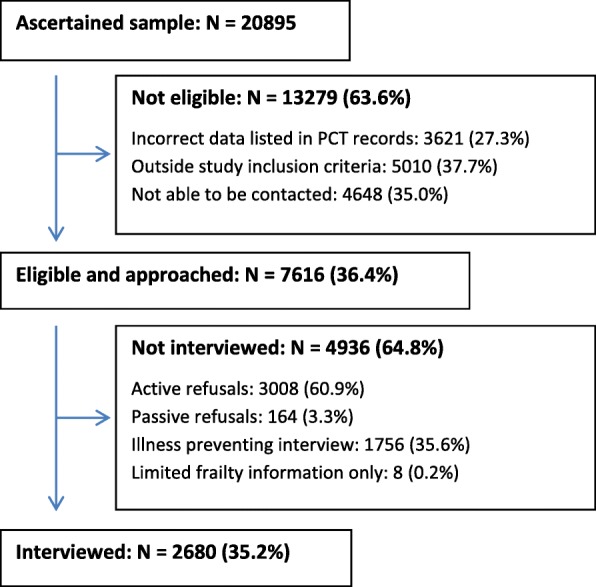
Flow of participants in Cam-CAN from sample to interview

Figure 1 illustrates the flow of participants in the Cam-CAN study from the ascertained sample to those interviewed (see Additional file 1 for detailed information of refusal reasons within each of the categories).

A large proportion of the ascertained sample (63.6%) were ineligible to participate due to reasons falling within the following three broad categories: incorrect data listed in PCT records (17.3%); factors outside the study inclusion criteria (24.0%) and being unable to be contacted (22.2%). Within older age groups, not being able to be contacted was the main reason for ineligibility. In younger age groups, ineligibility was mostly due to individuals having moved out of the area. Being a term time only student was a predominant ineligibility factor within the youngest age group. With Cambridge being a University City, such a dynamic population is expected with individuals moving and not re-registering elsewhere proving a major factor in recruitment.

The proportion of people who consented to participate out of those who were eligible and approached was 35.2%. Table 1 details a comparison between participants and non-participants of gender, age and Townsend deprivation index information. Overall, no evidence was found suggesting a difference in participation between men and women. However, an association was found between age and participation with middle age groups more likely to participate than younger and older age groups. An association between deprivation and participation was also found with the highest quintile of deprivation (most deprived) having the lowest proportion of individuals agreeing to participate (OR: 0.6, CI: 0.5–0.7).

**Table 1:** Descriptive statistics and associations between demographics and participation

	Not Interviewed (%) *N* = 4928	Interviewed (%) *N* = 2680	OR^a^	95% CI
Gender				
Men	2143 (43.5)	1172 (43.7)	1	–
Women	2785 (56.5)	1508 (56.3)	1.1	1.0–1.2
Age Group (years)				
18–27	516 (10.5)	204 (7.6)	0.9	0.7–1.1
28–37	745 (15.1)	364 (13.6)	1.1	0.9–1.3
38–47	590 (12.0)	347 (13.0)	1.3	1.1–1.5
48–57	299 (6.1)	251 (9.4)	1.8	1.5–2.2
58–67	373 (7.6)	359 (13.4)	2.1	1.7–2.4
68–77	545 (11.1)	398 (14.9)	1.6	1.3–1.9
78–87	1323 (26.9)	620 (23.1)	1	–
88+	537 (10.9)	137 (5.1)	0.5	0.4–0.7
Townsend Deprivation Index				
Quintile 1 (Least deprived)	925 (18.8)	576 (21.5)	0.9	0.8–1.1
Quintile 2	944 (19.2)	631 (23.5)	1	–
Quintile 3	939 (19.1)	548 (20.5)	0.9	0.8–1.0
Quintile 4	1009 (20.5)	482 (18.0)	0.8	0.6–0.9
Quintile 5 (Most deprived)	1111 (22.5)	443 (16.5)	0.6	0.5–0.7

^a^Odds ratio (OR) for participation and 95% Confidence Interval (CI), adjusted for all other factors in the table

An interaction between age and gender was identified (*p* = 0.01) with the relationship explored in depth in Table 2. In younger age groups, women were more likely to participate than men. However older age groups saw the opposite with men slightly more likely to participate. No evidence was found to suggest interactions between gender and deprivation or age and deprivation existed.

**Table 2:** Association for the interaction of age with gender on participation

	Men		Women	
Age Group	OR^a^	95% CI	OR	95% CI
18–27	0.7	0.5–0.9	1.0	0.7–1.3
28–37	0.8	0.7–1.1	1.2	1.0–1.5
38–47	1.1	0.8–1.4	1.3	1.0–1.7
48–57	1.7	1.2–2.2	1.7	1.3–2.2
58–67	1.8	1.4–2.4	2.0	1.5–2.5
68–77	1.6	1.3–2.0	1.3	1.0–1.7
78–87	1	–	0.9	0.7–1.1
88+	0.6	0.4–0.9	0.5	0.4–0.6

^a^Odds ratio (OR) for participation and 95% Confidence Interval (CI)

As previously described, reasons given for refusal were mapped into three broad categories: active refusals, passive refusals and refusals reflecting illness which prevented participation. Whilst most people were willing to express a reason for declining to take part, 20.3% of people did not wish to state a reason, with 14.1% of these reported by a proxy. For this analysis, no reason for refusal expressed directly by a participant counted as an active refusal, whilst proxy reported was treated as a passive refusal. The majority of refusals were active (61.0%), 3.3% passive with 35.6% deemed too ill to participate. The most common refusal reasons given within these categories are detailed in Table 3, with “too busy” as the most common active refusal, refusal by a relative for passive refusal and GP refusing a patient to be involved within the illness category.

**Table 3:** Most common reasons for refusal in active, passive and ill categories

Active refusals (61% of refusals)	*N*	% of Active
Too busy	1169	38.9
No reason given	860	28.6
Cannot be bothered	835	27.8
Passive refusals (3.3% of refusals)	*N*	% of Passive
Refusal by relative	102	62.2
Refusal by residential/nursing home	26	15.9
Not in after appointments made	23	14.0
Ill refusals (35.6% of refusals)	*N*	% of Ill
GP refusal of one patient	1115	63.5
Too ill (self reported)	337	19.2
Too ill (proxy reported)	149	8.5

Reasons given for refusal were analysed by gender, age and deprivation with results presented in Table 4. We found no gender effect for the type of refusal (active, passive or illness). Only a minor difference was found in reasons for refusal between the deprivation quintiles although this was explained by age and gender. Refusals within the illness category were more common in older age groups, however a high percentage of people in the youngest two age groups also refused for illness reasons. Active refusals were more common in middle age groups with the main reason given of being too busy.

**Table 4:** Refusal reasons explored by gender, age and deprivation

	Active		Passive		Ill	
	*N*	%	*N*	%	*N*	%
Gender						
Men	1370	63.9	66	3.1	707	33.0
Women	1638	58.8	98	3.5	1049	37.7
Age Group (years)						
18–27	350	67.8	18	3.5	148	28.7
28–37	561	75.3	15	2.0	169	22.7
38–47	469	79.5	15	2.5	106	18.0
48–57	246	82.3	11	3.7	42	14.1
58–67	291	78.0	5	1.3	77	20.6
68–77	361	66.2	19	3.5	165	30.3
78–87	587	44.4	43	3.3	693	52.4
88+	143	26.6	38	7.1	356	66.3
Townsend Deprivation Index						
Quintile 1 (Least deprived)	579	62.6	25	2.7	321	34.7
Quintile 2	585	62.0	40	4.2	319	33.8
Quintile 3	591	62.9	34	3.6	314	33.4
Quintile 4	562	55.7	31	3.1	416	41.2
Quintile 5 (Most deprived)	691	62.2	34	3.1	386	34.7

## Discussion

The Cam-CAN study has provided a unique opportunity to explore not only the pattern of response but also the demographic characteristics of individuals who either agree or disagree to participating in a large-scale epidemiological study. A large proportion of the ascertained sample (63.6%) was ineligible to be approached, with the eligibility criteria having different effects upon each age group. The highest participation was in the 58–67 years age group which includes recently retired individuals. In younger age groups, women were more likely to participate in comparison to men but the opposite was true in older age groups. Active refusals were given mostly by people in the middle age groups with the main reason being due to time constraints. Refusals due to illness were mostly seen in the oldest age groups.

One of the greatest strengths of the Cam-CAN study is that it is one of the very few true population based studies using neuroimaging to explore factors involved in ageing healthily across the entire adult age range. This does however bring with it difficulties in recruitment due to heavy participant burden with some individuals asked to complete three extensive data collection stages. It has been shown that the more a study requests of a potential participant, the more likely they are to refuse participation [5].

A strength of the study design was the way that potential participants were approached, such that both ‘opt-in’ and ‘opt-out’ recruitment strategies were employed. Individuals could actively agree or decline participation in the first instance after receiving the invitation letter (‘opt-in’). For individuals who remained passive, a research interviewer approached the individual at the contact address directly (‘opt-out’). Further investigation of the characteristics of recruited individuals by the two approach methods is planned. The method of approach was the same for all individuals regardless of their age so a direct comparison of refusal reasons can be performed [10].

Whilst most research studies do not cover the same age range as Cam-CAN, studies have reported highest participation rates are found among middle age groups in comparison with younger age groups [8, 10]. Consistent with our findings, there are reports that in older age groups, higher participation comes from age groups where individuals may have just retired [7, 10].

Findings from other studies have shown women are more willing to participate than men [5, 7, 13]. However, evidence from another population based investigation recently undertaken in the ageing population of England (Cognitive Function and Ageing Studies II, CFAS II) found men were more likely to participate [14]. In Cam-CAN, we observed a gender imbalance switching from being female dominated in younger age groups to more male orientated in older age groups. Other studies from the UK [10] and Denmark [15] also found an interaction between age and gender on non-response, although one only reported a change over the age of 70 years [10]. Such decreases in participation observed as age increases in women could be accounted for by the time demands modern society places on women who might be providing care for grandchildren [16].

The finding that increased deprivation is associated with non-participation is consistent with findings from other studies [15, 17, 18]. However, there seems little research into the interaction between deprivation and age upon participation, including the pattern we observed that deprivation becomes a more important factor within the older age groups.

There is an ever growing need to understand the reasons behind refusal with declining participation rates in epidemiological studies. It is important researchers are aware such biases exist and how they may impact study recruitment so adjustments and sensitivity analyses can be made accordingly. An increased awareness of refusal reasons could help to plan recruitment strategies leading to higher response rates.

## Conclusion

In conclusion, we explored demographic characteristics of participants and non-participants within the Cam-CAN cohort, along with reasons given for refusal. Whilst important to find age had an independent association with participation, it was interesting to observe its interplay with other demographic factors such as the interaction between age and gender. With age apparently having a different effect on response, perhaps more age-specific targeted recruitment strategies could be considered including the use of employing social media for recruiting younger individuals which has been found to be an effective recruitment tool [19].

## Additional file

Additional file 1: Figure detailing the flow of participants in Cam-CAN from sample to interview. (DOCX 37 kb)

## Abbreviations

Cam-CAN: Cambridge Centre for Ageing and Neuroscience
CFAS II: Cognitive Function and Ageing Studies II
GP: General Practitioner
PCT: Primary Care Trust
