# Supplementary material for: Exploring patterns of response across the lifespan: the Cambridge Centre for Ageing and Neuroscience (Cam-CAN) study
Source: BMC Public Health. 2018 Jun 19;18:760. doi: 10.1186/s12889-018-5663-7 (PMC6006958; doi:10.1186/s12889-018-5663-7)
Supplement: Supplementary file 1 — Figure detailing the flow of participants in Cam-CAN from sample to interview. (DOCX 37 kb) [file 12889_2018_5663_MOESM1_ESM.docx]

**Additional file 1:** Flow of participants in Cam-CAN from sample to interview in further detail

**Enumerated sample: N = 36018**

**Ascertained sample: N = 20895**

**Removal of 15123 individuals as follows:**

Oversampled individuals: 13637

Duplicated individuals: 1486

**Not eligible: N = 13279**

**Incorrect data listed in PCT records: N = 3621**

Not in correct area: 2524

Moved out of area before sampling date: 1021

Incorrect address: 62

Died before sampling date: 13

Incorrect date of birth: 1

**Outside study inclusion criteria: N = 5010**

GP surgery not used in year one: 3536

Term time only student: 1021

GP surgery not used in year two: 224

Language ineligibility: 176

GP declined to be involved: 39

Relative of co-applicant/co-investigator: 7

Study operation errors: 3

Profound deafness/unable to speak: 2

Participant participated in pilot investigation: 2

**Not able to be contacted: N = 4648**

Moved out of area before interview: 2288

Uncontactable after three attempts: 1113

Listed by PCT but no longer registered with GP: 1007

Died before interview: 190

Address not found, not a real address: 25

Not traced, patient does not exist: 20

Incorrect address listed by GP: 3

Uncontactable - no response to letters: 2

**Eligible and approached**

**N = 7616**

**Not interviewed: N = 4928**

**Active refusals N = 3008**

Too busy: 1169

No reason given: 860

Cannot be bothered: 835

Does not agree with surveys/science: 84

Fear of Magnetic Resonance Imaging: 16

I have done enough: 16

Knows someone who has been interviewed: 12

Respondent requested data be removed: 8

Not worth time for payment: 3

Unreliable/time waster: 3

Refusal of a specific test: 2

**Passive refusals N = 164**

Refusal by relative: 102

Refusal by residential/nursing home: 26

Not in after appointments made: 23

Refusal by neighbour: 13

**Illness preventing interview N = 1756**

GP refusal – one patient: 1115

Too ill – self report: 337

Too ill – proxy report: 149

Nervous/wary: 75

GP refusal – too ill: 74

Demonstrated inappropriate behaviour: 6

**Limited frailty information only: N = 8**

Informant only interview – informant refused: 7

Informant only interview – no informant identified: 1

**Interviewed: N = 2680**

HAS interview: 4

Subject interviews: 2676
